# Quantitative sonographic assessment of quadriceps muscle thickness for fall injury prediction in patients undergoing maintenance hemodialysis: an observational cohort study

**DOI:** 10.1186/s12882-021-02347-5

**Published:** 2021-05-22

**Authors:** Asuka Sai, Kentaro Tanaka, Yasushi Ohashi, Akifumi Kushiyama, Yoshihide Tanaka, Shuta Motonishi, Ken Sakai, Shigeko Hara, Takashi Ozawa

**Affiliations:** 1 Kodaira Kitaguchi Clinic, Tokyo, Japan; 2 Higashikurume Ekimae Clinic, Tokyo, Japan; 3 Department of Nephrology, Sakura Medical Center, Toho University, 564-1, Shimoshizu, Sakura, Chiba, 285-8741 Japan; 4 The Division of Diabetes and Metabolism, The Institute for Adult Diseases, Asahi Life Foundation, Tokyo, Japan; 5 Department of Pharmacotherapy, Meiji Pharmaceutical University, Tokyo, Japan; 6 Kumegawa tousekinaika Clinic, Tokyo, Japan; 7 Higashiyamato Nangai Clinic, Tokyo, Japan; 8 Department of Nephrology, Faculty of Medicine, Toho University, Tokyo, Japan; 9 Okinaka Memorial Institute for Medical Research, Toranomon Hospital, Tokyo, Japan

**Keywords:** Injury, Falls, Hemodialysis, Quadriceps thickness, Sarcopenia, Ultrasonography

## Abstract

**Background:**

Accidental fall risk is high in patients undergoing maintenance hemodialysis. Falls are associated with fatal injury, comorbidities, and mortality. Risk assessment should be a primary component of fall prevention. This study investigated whether quadriceps muscle thickness measured using ultrasonography can predict fall injury among dialysis patients.

**Methods:**

Using an observational cohort study design, 180 ambulatory hemodialysis patients were recruited from 2015 to 2016 from four dialysis clinics. The sum of the maximum quadriceps muscle thickness on both sides and the average of the maximum thigh circumference and handgrip strength after hemodialysis were calculated. Patients were stratified according to tertiles of quadriceps muscle thickness. Fall injury was surveyed according to the patient’s self-report during the one-year period.

**Results:**

Among the 180 hemodialysis patients, 44 (24.4%) had fall injuries during the 12-month follow-up period. When the quadriceps muscle thickness levels were stratified into sex-specific tertiles, patients in the lowest tertile were more likely to have a higher incidence of fall injury than those in the higher two tertiles (0.52 vs. 0.19 and 0.17 fall injuries/person-year). After adjusting for covariates, lower quadriceps muscle thickness was found to be an independent predictor of fall injury (hazard ratio [95% confidence interval], 2.33 [1.22–4.52], *P* < 0.05). Receiver operating characteristic curves were constructed to determine the optimal cutoffs of quadriceps muscle thickness, thigh circumference, and handgrip strength that best predicted fall injury (quadriceps muscle thickness, 3.37 cm and 3.54 cm in men and women; thigh circumference, 44.6 cm and 37.2 cm in men and women; and handgrip strength, 23.3 kg and 16.5 kg in men and women). Using these cutoff values, the areas under the curve were 0.662 (95% CI, 0.576–0.738), 0.625 (95% CI, 0.545–0.699), and 0.701 (95% CI, 0.617–0.774), for quadriceps muscle thickness, thigh circumference, and handgrip strength, respectively. Quadriceps muscle thickness was a more precise predictor of fall injury than thigh circumference and had similar diagnostic performance as handgrip strength tests in dialysis patients.

**Conclusions:**

Quadriceps muscle thickness can be measured easily at the bedside using ultrasonography and is a precise predictor of fall injury in patients undergoing maintenance hemodialysis.

**Supplementary Information:**

The online version contains supplementary material available at 10.1186/s12882-021-02347-5.

## Background

The elderly population is rapidly increasing in Japan. According to a statistical survey conducted by the Japanese Society for Dialysis Therapy at the end of 2016, the mean age of patients on dialysis is 67.2 years [1]. As the aging trend continues, the incidence of fall injury is likely to increase. Falls as a cause of fractures, may lead to the deterioration of the physical condition in elderly patients. Surprisingly, fall accidents occur in approximately 25% of maintenance hemodialysis patients each year [2]. Approximately 20–30% of these patients experience mild to severe injury, while more than half of these patients are hospitalized, which results in high health system costs. Therefore, the establishment of risk assessments for falls and countermeasures are urgent issues, with risk assessment as a primary component of fall prevention.

Sarcopenia, characterized by age-related decreases in skeletal muscle mass and muscle strength and low physical performance, is a geriatric syndrome that has been recognized in elderly care during the last decade. A common sarcopenia consensus was published by the European Working Group on Sarcopenia in Older People (EWGSOP) in 2010 [3] and by the Asian Working Group for Sarcopenia (AWGS) in 2014 [4]. Sarcopenia increases the risks for adverse health outcomes such as falls, physical disability, hospital admission, poor quality of life, and mortality risk [5, 6]. In fact, hemodialysis patients have high risks for falls not only because of aging but also because of unstable hemodynamic status or some comorbidities, and falls are associated with increased mortality risk among these patients [7, 8]. The EWGSOP recommends that muscle mass should be measured using computed tomography (CT) scans and magnetic resonance imaging (MRI) as the gold standard. However, these methods are costly, and most hemodialysis clinics cannot provide such medical facilities [2]. Conversely, ultrasonography is widely available, noninvasive, and easily applicable at the bedside for the quantitative assessment of skeletal muscle. The measurement of quadriceps muscle thickness using ultrasonography may be useful for muscle mass assessment, which may help predict future fall injury.

This study aimed to investigate the validity of quadriceps muscle thickness measurement conducted noninvasively using ultrasonography and the association of quadriceps muscle thickness with future fall injury among maintenance hemodialysis patients.

## Methods

### Participants and study design

The subjects were recruited from a pool of 629 enrolled outpatients aged ≥20 years old with end-stage renal disease undergoing maintenance hemodialysis in daytime sessions three times per week for more than 3 months at the four dialysis clinics in April 2015 (403 men and 226 women; median age [interquartile range], 71 [65–79] years). This study was approved by the Institutional Ethics Committee of Medical Toyou, Japan (No. 2014–7), and written informed consent was obtained from 189 patients. We excluded patients with physical disability (*n* = 7) or who experienced cardiovascular events within 1 month (*n* = 2). Consequently, 180 patients were included in the study. Using a prospective cohort study design, the frequency of fall injury was recorded based on the patient’s self-report during a follow-up period of 12 months from April 2015 to March 2016 (median days, 365 days [275–365 days]). A fall was defined as an event in which a person was inadvertently located on the ground or another low position without any symptoms of postdialysis hypotension, such as dizziness, weakness, or disturbance of consciousness. Fall injury was defined as any injury associated with a fall, including bone fracture, cracks, bleeding, bruising, and abrasion.

The surveyed subject characteristics included age, sex, anthropometric measures, underlying renal disease, hemodialysis duration (3.5 h, 4.0 h, or > 4 h), fluid removal volume, use of antihypertensive and benzodiazepine drugs, and any immediate management for intradialytic hypotension, such as cessation of ultrafiltration, Trendelenburg position, saline infusion, reduction in blood flow, and presence or absence of intradialytic hypotension. Intradialytic hypotension was defined as a decrease in systolic blood pressure of ≥20 mmHg or a decrease in mean arterial pressure of 10 mmHg that was associated with symptoms including abdominal discomfort, yawning, sighing, nausea, vomiting, muscle cramps, restlessness, dizziness, fainting, or anxiety [9]. The following data were extracted: intact parathyroid hormone, serum albumin, lipid profile, uric acid, c-reactive protein, blood urea nitrogen, creatinine, calcium, phosphorus, β_2_ microglobulin, and hemoglobin levels. Blood tests were performed at the start of dialysis during the first day of the week. Dialysis adequacy assessed in terms of the urea reduction ratio and single pool Kt/V was measured using the Shinzato formula [10].

### Measurements of quadriceps muscle thickness, thigh circumference, and handgrip strength

**Fig. 1:**
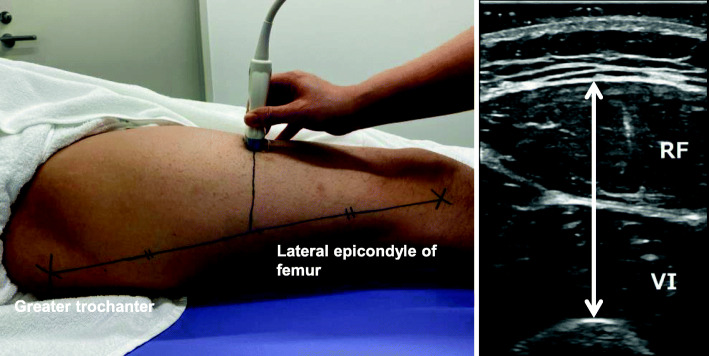
Measurement of the quadriceps muscle thickness. Abbreviations: RF, rectus femoris; VI, vastus intermedius muscle; QT, quadriceps muscle thickness. The QT, which consists of the thickness of the rectus femoris and the vastus intermedius muscle, was measured at a distance of 50% between the greater trochanter and the lateral epicondyle of the femur. Using a B-mode ultrasound apparatus (LOGIQ BOOK XP; GE Healthcare Japan, Tokyo, Japan) with a linear scanner, the procedure was conducted by a single examiner with the patient in the supine position after dialysis

We measured the rectus femoris and the vastus intermedius muscle by a B-mode ultrasound apparatus (LOGIQ BOOK XP; GE Healthcare Japan, Tokyo, Japan) to evaluate quadriceps muscle thickness. As some patients could not fully extend their knee, a single experienced examiner placed a 6–12 MHz linear transducer perpendicularly to the front of the thigh at a distance of 50% between the greater trochanter and the lateral epicondyle of the femur with excessive gel to reduce pressure on the quadriceps muscle (Fig. 1) [11]. After dialysis, patients lay in the supine position, with the limb extended within the realms of possibility. The thickness of each side of the quadriceps muscle was measured twice, and the sum of the larger value for each side was used.

We also measured the maximum thigh circumference on both sides and calculated the average. The handgrip strength was measured before dialysis using a hand-held dynamometer (Takei Scientific Instruments Co, Niigata Japan) with the participants in a sitting position with their elbow fully extended and their arm parallel to the ground. Before testing, a technician demonstrated proper form and emphasized a quick and hard squeeze of the handle with maximal effort. The timing of the 2nd measurement was entrusted to the participants by themselves whenever muscle fatigue was recovered after a short break. The highest grip strength after maximal effort was recorded for the right and left sides alternately two times, and the maximum value was adopted.

### Statistical analysis

All data are expressed as the median and interquartile range for continuous variables and as the number and percentage for categorical variables. The Shapiro-Wilks test was used to determine whether this study sample has been drawn from a normally distributed population. The statistical significance was analyzed by the non-parametric tests. The differences between the two groups were assessed using the Mann–Whitney U test, and the differences between the matched pairs were assessed using the Wilcoxon signed-ranks test. The differences among the tertile groups for continuous variables were also assessed using the Kruskal-Wallis test. If there were significant differences in the overall group, the differences in each group were assessed using the Steel-Dwass test. The differences among the tertile groups for categorical variables were also assessed using Pearson’s chi-squared test. To investigate the relationship between the quadriceps muscle thickness levels and the first onset of fall injury, the quadriceps muscle thickness levels were stratified into tertiles, which were categorized separately for men and women. Fall injury as the primary outcome was analyzed using the Kaplan–Meier method, and significance was calculated using the log-rank test. The Cox proportional hazard models were performed for fall injury and patient-related risk factors, such as quadriceps muscle thickness levels, age (1 year increments), sex (men vs. women), diabetes (presence vs. absence), stroke (presence vs. absence), serum albumin (g/dl), blood urea nitrogen (mg/dl), uric acid (mg/dl), and grip strength (kg). These results are expressed as hazard ratios with 95% confidence intervals (CIs). Receiver operating characteristic curve analysis was used to identify the best prognostic factor for fall injury. *P*-values < 0.05 were considered to indicate statistically significant differences. All data were analyzed using JMP software (version 13.0; SAS Institute Cary, NC, USA).

## Results

### Study population

A total of 180 patients were included, with a total of 127 men and 53 women (median age, 69 [63–76] years old; median duration of dialysis, 5.5 [2.4–9.7] years). The median sum of quadriceps muscle thickness, the median thigh circumference, and the median handgrip strength in men were 4.0 (3.4–4.9) cm, 43.0 (40.5–46.5) cm, and 26.1 (22.1–32.1) kg, respectively. These values in women were 4.0 (3.3–4.9) cm, 40.0 (37.0–46.0) cm, and 17.1 (14.9–21.6) kg, respectively. There were significant differences between men and women in thigh circumference and handgrip strength, and the circumference and strength of the right sides were more likely to be wider and stronger than those of the left sides. In contrast, these differences were not observed in quadriceps muscle thickness (Additional Table 1). This sample was presented non-normally distributed population in quadriceps muscle thickness and in thigh circumference and was presented normally distributed population in handgrip strength. The relative reliability of quadriceps muscle thickness measured by ultrasonography was confirmed using the intraclass correlation coefficient (ICC) (right quadriceps muscle thickness ICC (1,2) = 0.99 and left quadriceps muscle thickness ICC (1,2) = 0.98).

During a 12-month follow-up period, 44 (24.4%) out of those 180 patients had fall injuries; 19 and 25 had fallen on dialysis days and on no dialysis days, respectively. Patients with fall injury were more likely to be diabetes, history of stroke, and history of ischemic heart disease, and those were more likely to have lower creatinine, quadriceps muscle thickness, thigh circumference and handgrip strength (Additional Table 2).

The baseline clinical and biochemical characteristics of the quadriceps muscle thickness tertiles are shown in Table 1. (the lowest tertile: < 3.66 cm; the middle tertile: 3.66–4.59 cm; the highest tertile: ≥4.60 cm in men and the lowest tertile: < 3.50 cm; the middle tertile: 3.50–4.49 cm; the highest tertile: ≥4.50 cm in women). The individuals in the lowest quadriceps muscle thickness tertile were significantly older than those in the higher two tertiles. These patients were more likely to have lower body mass index (BMI) and uric acid and serum creatinine levels than those in the higher two tertiles and tended to have lower fluid volume removal, triglyceride and serum phosphate levels than those in the highest tertile. Moreover, thigh circumference and handgrip strength were significantly lower in the tertile with the lowest quadriceps muscle thickness. Patients with hemodialysis duration > 4.0 h were more likely to be in the highest tertile. Patients taking fewer than 2 antihypertensive drugs were more likely to be in the lowest tertile.

**Table 1:** Baseline characteristics of the subjects stratified by tertiles of quadriceps muscle thickness

Sample characteristics	Quadriceps muscle thickness measured using ultrasonography			*P*-value
	Tertile 1 (n = 60, < 3.66 cm in 42 men and < 3.50 cm in 18 women)	Tertile 2 (*n* = 60, 3.66–4.59 cm in 43 men and 3.50–4.49 cm in 17 women)	Tertile 3 (n = 60, ≥4.60 cm in 42 men and ≥ 4.50 cm in 18 women)	
Age (years)	74 (67–80)*^#^	67 (65–74)*	66 (50–72)	< 0.001
Duration of dialysis (years)	5.1 (2.3–8.7)	5.4 (2.3–9.7)	6.4 (2.9–11.3)	0.47
Body mass index (kg/m^2^)	19.9 (18.4–21.7)*^#^	20.9 (19.8–23.8)*	25.0 (22.1–26.9)	< 0.001
Follow-up period (days)	315 (171–365)*^#^	365 (348–365)	365 (365–365)	< 0.001
Dialysis prescription				
HD duration of 3.5 h/4.0 h/> 4.0 h, n	1 / 59 / 0	0 / 55 / 5	2 / 45 / 13	< 0.001
Fluid volume removal (L)	2.2 (1.8–2.8)*	2.4 (1.9–3.0)*	3.0 (2.3–3.3)	< 0.001
Antihypertensive drugs, 0, 1, 2, or 3 or more, n	10 / 20 /11 / 19	14 / 4 / 16 / 26	13 / 5 / 25 / 17	0.032
Benzodiazepine drugs, n (%)	11 (18)	10 (17)	15 (25)	0.80
Any treatments for intradialytic hypotension, n (%)	15 (25)	10 (17)	11 (18)	0.80
Comorbidities				
Diabetes mellitus, n (%)	30 (50)	33 (55)	29 (48)	0.75
Stroke, n (%)	11 (18)	13 (22)	10 (17)	0.78
Ischemic heart disease, n (%)	20 (33)	25 (42)	22 (37)	0.64
Serum albumin (g/dl)	3.7 (3.4–3.9)	3.7 (3.6–3.9)	3.8 (3.6–4.0)	0.05
HDL-cholesterol (mg/dl)	43 (36–54)	41 (36–51)	40 (29–53)	0.23
LDL-cholesterol (mg/dl)	77 (59–97)	81 (63–98)	79 (65–102)	0.73
Triglyceride (mg/dl)	71 (58–117)*	92 (65–126)	106 (65–172)	0.012
Uric acid (mg/dl)	6.3 (5.4–7.0)*^#^	7.0 (5.9–7.9)	7.4 (6.3–8.3)	< 0.001
CRP (mg/dl)	0.09 (0.05–0.38)	0.11 (0.05–0.21)	0.13 (0.05–0.31)	0.88
BUN (mg/dl)	62 (50–74)	64 (55–78)	65 (59–75)	0.40
Creatinine (mg/dl)	9.3 (8.1–10.6)*^#^	10.8 (9.3–13.1)*	12.4 (10.4–13.8)	< 0.001
Single pool Kt/Vurea	1.46 (1.36–1.61)	1.45 (1.36–1.63)	1.43 (1.31–1.57)	0.36
Ca (mg/dl)	8.7 (8.2–9.1)	8.7 (8.3–9.0)	8.9 (8.5–9.3)	0.08
P (mg/dl)	5.1 (4.2–5.8)^#^	5.6 (5.1–6.5)	5.5 (4.7–6.2)	0.007
Casual blood glucose, (mg/dl)	135 (112–180)	126 (103–176)	122 (104–149)	0.45
Intact PTH (pg/ml)	136 (80–200)	150 (76–235)	128 (71–183)	0.37
β_2_ microglobin (mg/l)	25.4 (22.4–28.5)	27.5 (23.9–30.8)	26.7 (22.2–29.2)	0.12
Hemoglobin (g/dl)	11.0 (10.2–11.3)	10.7 (10.3–11.1)	10.8 (10.3–11.5)	0.75
Thigh circumference (cm)	40 (37–42)*^#^	43 (40–45)*	47 (44–51)	< 0.001
Handgrip strength (kg)	20.8 (16.4–25.2)*^#^	23.9 (20.6–28.8)	25.3 (21.5–33.0)	< 0.001

Abbreviations: *n* number; *h* hours; *HDL* high-density lipoprotein; *LDL* low-density lipoprotein; *CRP* C-reactive protein; *BUN* blood urea nitrogen; *PTH* parathyroid hormone

Data are expressed as the median (interquartile range) or number (percentage)

*P < 0.05, compared with tertile 3; ^#^*P* < 0.05, compared with tertile 2

### Associations of quadriceps muscle thickness, thigh circumference, and handgrip strength with fall injury

**Fig. 2:**
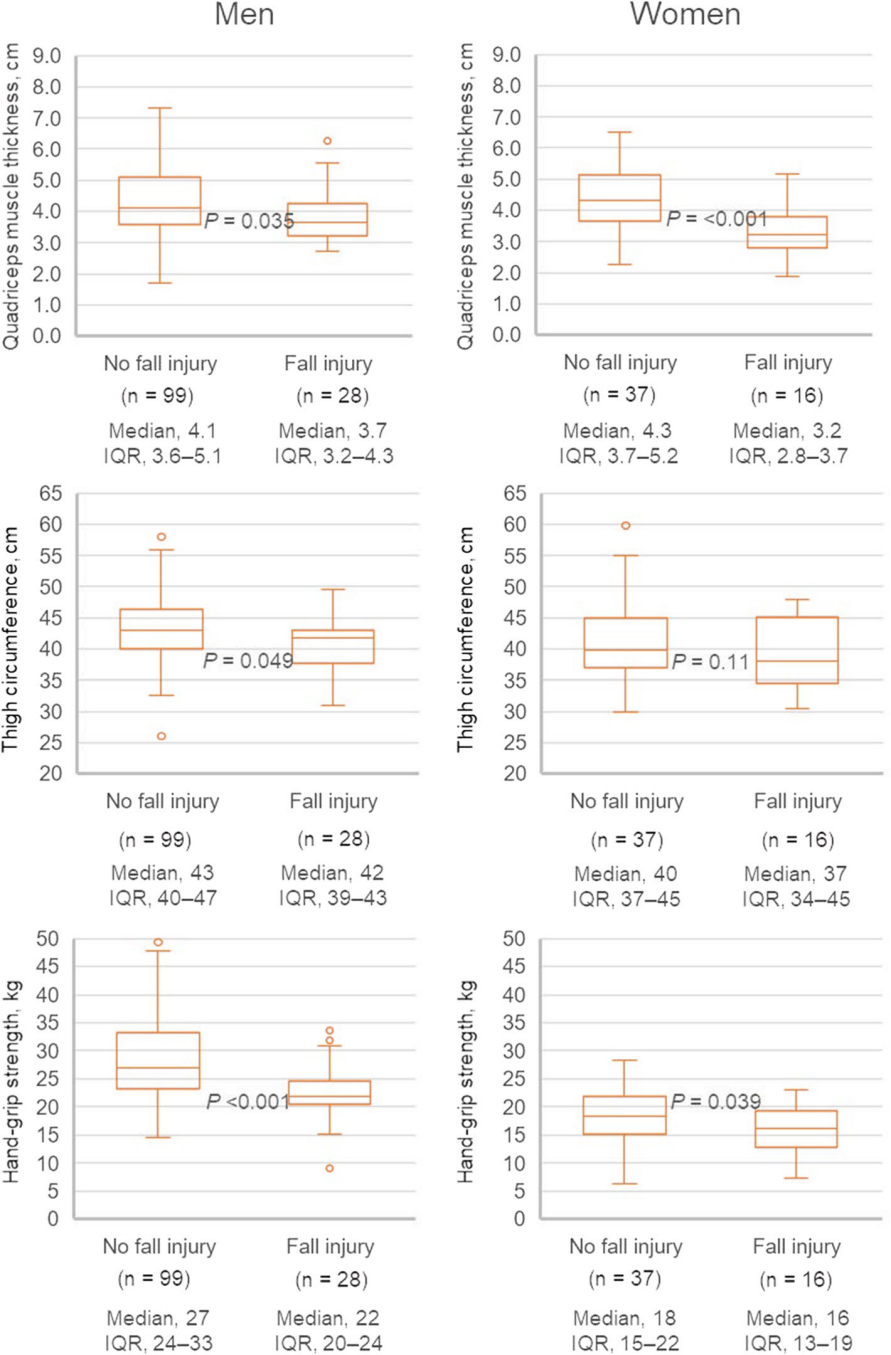
Quadriceps muscle thickness, thigh circumference, and handgrip strength in patients with and without fall injury

As shown in Fig. 2, men with fall injury were more likely to have lower quadriceps muscle thickness, thigh circumference, and handgrip strength than men without fall injury. Women with fall injury were also more likely to have lower quadriceps muscle thickness and handgrip strength than women without fall injury. Thigh circumference tended to be lower in women with fall injury than in women without fall injury. However, no significant differences were observed among these values.

**Fig. 3:**
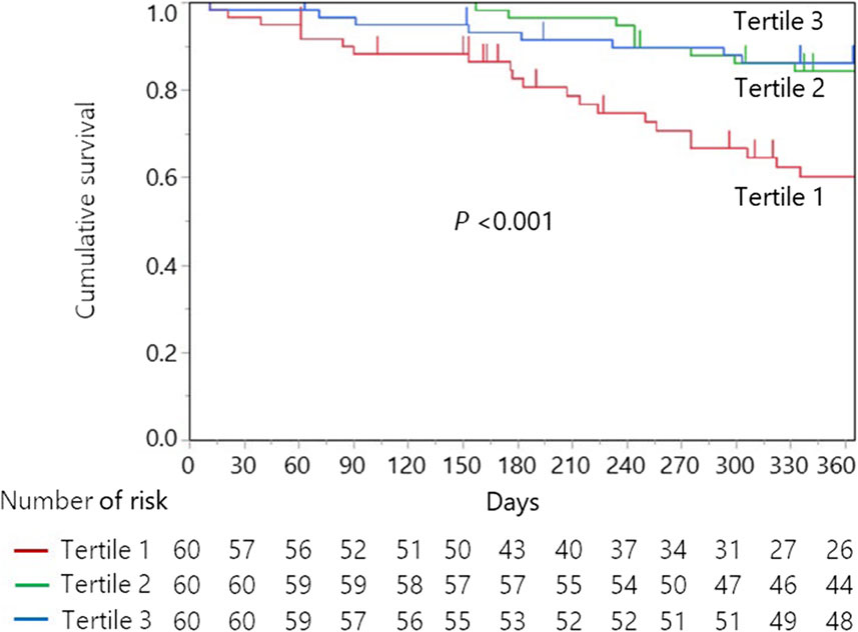
Kaplan–Meier curves for fall injury by tertiles of quadriceps muscle thickness

Patients in the lowest tertile had a significantly higher risk of fall injury than those in the higher two tertiles (log-rank test, *P* < 0.001) (Fig. 3). Individuals in the lowest tertile had 25 events, an incidence of 0.57 fall injuries/person-year, and a cumulative incidence of 48.7%. The middle tertile and the highest tertile had almost the same frequency of fall injury, which was lower than that in the lowest tertile. The middle tertile developed 11 events (an incidence of 0.19 fall injuries/person-years, 19.4% cumulative incidence), and the highest tertile developed 8 events (an incidence of 0.17 fall injuries/person-years, 13.8% cumulative incidence).

In univariate analysis, the lowest quadriceps muscle thickness tertile, diabetes, history of stroke, history of ischemic heart disease, albumin, blood urea nitrogen, uric acid, and handgrip strength were associated with a higher risk of fall injury. In multivariate analysis, the association of the lowest quadriceps muscle thickness tertile with fall injury risk remained significant after covariates were adjusted (hazard ratio [95% CI], 2.33 [1.22–4.52], *P* < 0.001) (Table 2). Creatinine and thigh circumference were not included as cofounding factors between quadriceps muscle thickness and fall injury.

**Table 2:** Hazard risks of quadriceps muscle thickness on fall injury

Variables	Hazard ratio (95% CI)	*P*-value
Unadjusted	3.55 (1.96–6.54)	< 0.001
Age- and gender- adjusted	3.33 (1.72–6.59)	< 0.001
Multivariable-adjusted^a^	2.33 (1.22–4.52)	0.011

Abbreviation: *CI* confidence interval

Data are expressed as hazard ratios with 95% confidence intervals

^a^Adjusted for diabetes mellitus, history of stroke, history of ischemic heart disease, albumin, blood urea nitrogen, uric acid, and handgrip strength (variables associated with fall injury in univariate analysis (*P* < 0.10) were entered in the multivariable model). Creatinine and thigh circumference were not included as cofounding factors in the association between quadriceps muscle thickness and fall injury

### Predictive performance of quadriceps muscle thickness, thigh circumference, and handgrip strength for fall injury

Receiver operating characteristic curves were constructed to determine the optimal cutoffs of quadriceps muscle thickness, thigh circumference, and handgrip strength that best predicted fall injury (quadriceps muscle thickness, 3.37 cm and 3.54 cm in men and women; thigh circumference, 44.6 cm and 37.2 cm in men and women; and handgrip strength, 23.3 kg and 16.5 kg in men and women). Using these cutoff values, the areas under the curve were 0.662 (95% CI, 0.576–0.738), 0.625 (95% CI, 0.545–0.699), and 0.701 (95% CI, 0.617–0.774), respectively. As shown in Table 3, the sensitivity of quadriceps muscle thickness for fall injury was lower than that of the other two modalities (50% vs. 75.0 and 68.2%). On the other hand, the specificity of quadriceps muscle thickness for fall injury was the highest of these three modalities (82.4% vs. 50.0 and 72.1%). Consequently, the false positive rate of quadriceps muscle thickness was the lowest of these three modalities (17.6% vs. 50.0 and 27.9%). Interestingly, fall injuries in patients with quadriceps muscle thickness or thigh circumference below the cutoff values were observed earlier within a 60-day period, and these findings were not observed in patients with handgrip strength below the cutoff values (Additional Figure).

**Table 3:** Predictive performance of quadriceps muscle thickness, thigh circumference, and handgrip strength for fall injury

Predictive values	Quadriceps muscle thickness (cutoff values of 3.37 cm in men and 3.54 cm in women)	Thigh circumference (cutoff values of 44.6 cm in men and 37.2 cm in women)	Handgrip strength (cutoff values of 23.3 g in men and 16.5 g in women)
Sensitivity, n (%)	22/44 (50.0)	33/44 (75.0)	30/44 (68.2)
False positive rate, n (%)	24/136 (17.6)	68/136 (50.0)	38/136 (27.9)
Specificity, n (%)	112/136 (82.4)	68/136 (50.0)	98/136 (72.1)
False negative value, n (%)	22/44 (50.0)	11/44 (25.0)	14/44 (31.8)
Positive predictive value, n (%)	22/46 (47.8)	33/101 (32.7)	30/68 (44.1)
Negative predictive value, n (%)	112/134 (83.6)	69/82 (86.1)	98/112 (87.5)
Positive likelihood ratio	2.84	1.50	2.44
Negative likelihood ratio	0.61	0.50	0.44

Abbreviations: *n* number

## Discussion

In this cohort study, fall accidents were observed in 44 (24.4%) maintenance dialysis patients during a one-year period. The quadriceps muscle thickness measured using ultrasonography was significantly associated with future fall injury, with optimal cutoff values of 3.37 cm and 3.54 cm for men and women, respectively. The sensitivity and specificity of quadriceps muscle thickness were 50 and 82.4%, respectively. The false positive rate of quadriceps muscle thickness was 17.6%, which indicates that quadriceps muscle thickness was a more precise predictor of fall injury than thigh circumference and had a comparable diagnostic performance as handgrip strength tests in dialysis patients. Interestingly, fall injuries occurring earlier within a 60-day period were observed in patients with quadriceps muscle thickness or thigh circumference below the cutoff values. These findings were not observed in patients with handgrip strength below the cutoff values.

Falls tend to occur in elderly people and are commonly observed in dialysis patients. Fall injury often leads to hospitalizations, starting the downward spiral of physical disorder that can result in long-term functional disability or death. Muscle weakness and frailty are considered the most important risk factors for falls [4, 12]. Sarcopenia occurs more frequently in hemodialysis patients [5, 13]. The EWGSOP provided a common consensus for muscle mass assessment in aging populations. CT scans and MRI, which are the gold standard for precisely measuring skeletal muscle mass, are widely used. On the other hand, these techniques have numerous problems, including costs, limited number of facilities, time requirement, and radiation exposure, so most dialysis units cannot use these techniques routinely for a large number of subjects in general practice. Alternatively, dual-energy X-ray absorptiometry (DEXA) and bioelectrical impedance spectroscopy (BIA) are simpler inspection methods. However, DEXA remains problematic regarding radiation exposure and the inspection of equipment. Portable BIA may have calculation errors because excess fluid is miscalculated as muscle mass [14].

Recently, some studies reported on the reliability of bedside ultrasound for the measurement of muscle thickness [15–18]. Muscle thickness measured using a sonographic technique has a high correlation with the CT scan and MRI values [19]. Sabatino et al. [20] reported a significant decrease in quadriceps femoris muscle in hemodialysis patients compared to that in healthy adults. In this study, the quadriceps muscle thickness was comparable to the result of the previous study. More importantly, the quadriceps muscle thickness measured using ultrasonography was clinically verified as an independent risk factor for fall injury.

The following risk factors have been proposed for fall injury in hemodialysis patients: age, diabetes, handgrip strength, antidepressant agents, and sarcopenia [21, 22]. The association of diabetes with fall injury has already been investigated by several researchers [23, 24]. Diabetes patients are prone to fall due to complications such as hypoesthesia from peripheral neuropathy, loss of vision from retinopathy, orthostatic hypotension from autonomic disturbance, and hypoglycemia. In this study, diabetes was also associated with fall injury, which is similar to the findings of previous studies.

Low handgrip strength can possibly be a risk for falls or activities of daily living disability [25]. Handgrip strength is known to correlate with limb muscle strength and is an easy method for the evaluation of muscle strength [26]. In this study, handgrip strength was also associated with fall injury and had the highest diagnostic performance among the three modalities. However, the median handgrip strengths in women with fall injury and women without fall injury were 16 kg and 18 kg, respectively. The difference may hardly be distinguishable. Interestingly, handgrip strength was not associated with fall injuries occurring within 60 days, which may indicate that handgrip strength is indirectly associated with fall injuries by reflecting physical weakness with wasting. Muscle strength does not always depend on muscle mass; a study showed that the association between muscle strength and muscle mass is not linear [27].

Previous studies have confirmed that sarcopenia can lead to falls, disability, hospital admission, long-term care requirement, poorer quality of life, and increased mortality rate [26, 28]. Sarcopenia patients were found to be over three times more likely to fall than nonsarcopenia patients [29]. Japan is one of the most rapidly aging countries in the world. Moreover, hemodialysis patients are generally considered a high-risk group for sarcopenia due to inflammation, malnutrition associated with dietary therapy, loss of protein from dialysis membrane, low performance due to complications or comorbidity, and time loss due to dialysis schedules [30, 31]. Mainly, this study aimed to assess the risk of falls affecting the prognosis of hemodialysis patients since muscle thickness measured using ultrasonography has been confirmed to easily and accurately evaluate the risk of future fall injury.

This study had several limitations. The first limitation is the relatively small number of subjects enrolled in this study. We recognize that it is unclear whether the 180 patients included in the study are representative of the 629 identified outpatients aged ≥20 years old with end-stage renal disease undergoing maintenance hemodialysis in daytime sessions. At least there was no difference in age between the 180 participants and the 449 other patients. Moreover, the limited number of female participants may have led to the finding of a smaller association of thigh circumference with fall injury than those of the other two modalities. The second limitation is the measurement of quadriceps muscle thickness. Some patients could not hold the required position for ultrasonography because they could not fully extend their knee. Therefore, we modified the commonly used method [15, 17]. Third, the results were not compared with an observation of muscle mass using CT or MRI. Fourth, this study did not discuss the following issues: how to improve physical performance, quality of life, mortality from rehabilitation or nutrition management perspectives. Hence, further study is necessary to comprehensively consider methods of recuperation, including the abovementioned issues.

## Conclusions

Quadriceps muscle thickness can be easily measured at the bedside using ultrasonography and is a precise predictor of fall injury in patients undergoing maintenance hemodialysis. Thus, quantitative sonographic assessment of quadriceps muscle thickness can be worth considering from a prognostic point of view.

## Supplementary Information

**Additional file 1: Table 1.** Sample quadriceps muscle thickness, thigh circumference, and handgrip strength by sex. **Table 2.** Sample characteristics by absence or presence of fall injury.

**Additional file 2: Figure.** Kaplan–Meier curves for fall injury in quadriceps muscle thickness, thigh circumference, and handgrip strength.

## Data Availability

The datasets used and/or analyzed during the current study are available from the corresponding author on reasonable request.

## Abbreviations

EWGSOP: European Working Group on Sarcopenia in Older People
AWGS: Asian Working Group for Sarcopenia
CT: Computed tomography
MRI: Magnetic resonance imaging
ICC: Intraclass correlation coefficient
BMI: Body mass index
DEXA: Dual-energy X-ray absorptiometry
BIA: Bioelectrical impedance spectroscopy
