# Supplementary material for: Quantitative sonographic assessment of quadriceps muscle thickness for fall injury prediction in patients undergoing maintenance hemodialysis: an observational cohort study
Source: BMC Nephrol. 2021 May 22;22:191. doi: 10.1186/s12882-021-02347-5 (PMC8140437; doi:10.1186/s12882-021-02347-5)
Supplement: Supplementary file 1 — Additional file 1: Table 1. Sample quadriceps muscle thickness, thigh circumference, and handgrip strength by sex. Table 2. Sample characteristics by absence or presence of fall injury. [file 12882_2021_2347_MOESM1_ESM.docx]

# Additional Table 1. Sample quadriceps muscle thickness, thigh circumference, and handgrip strength by sex

| Measurements | Men  (n = 127) | Women  (n = 53) | *P*-value |
| --- | --- | --- | --- |
| Quadriceps muscle thickness |  |  |  |
| Median on right side, cm | 2.0 (1.7–2.5) | 2.0 (1.0–3.6) | 0.57 |
| Median on left side, cm | 2.0 (1.7–2.5) | 2.0 (1.6–2.3) | 0.48 |
| Median of average value on both sides, cm | 2.0 (1.7–2.5) | 2.0 (1.6–2.5) | 0.50 |
| Median of sum value on both sides, cm | 4.0 (3.4–4.9) | 4.0 (3.3–4.9) | 0.49 |
| Thigh circumference |  |  |  |
| Median on right side, cm | 43 (40–46)^a^ | 40 (37–46)^b^ | 0.020 |
| Median on left side, cm | 42 (40–46) | 40 (37–45) | 0.019 |
| Median of average value on both sides, cm | 43 (40–46) | 40 (37–45) | 0.018 |
| Median of maximum value on both sides, cm | 43 (41–47) | 40 (37–46) | 0.014 |
| Handgrip strength |  |  |  |
| Median of value for the 1st time on right side, kg | 25 (20–31) | 16 (13–21) | <0.001 |
| Median of value for the 2nd time on right side, kg | 25 (20–31) | 16 (13–20) | <0.001 |
| Median of maximum value on right side, kg | 26 (21–32)^c^ | 17 (14–21)^d^ | <0.001 |
| Median of value for the 1st time on left side, kg | 24 (19–28) | 15 (11–19) | <0.001 |
| Median of value for the 2nd time on left side, kg | 23 (19–28) | 15 (12–18) | <0.001 |
| Median of maximum value on left side, kg | 24 (20–29) | 16 (12–19) | <0.001 |
| Median of average value on both sides, kg | 25 (21–30) | 17 (13–20) | <0.001 |
| Median of maximum value on both sides, kg | 26 (22–32) | 17 (15–22) | <0.001 |

Data are expressed as median (interquartile range)

^a^*P*<0.05, right thigh circumference vs. left thigh circumference in men; ^b^*P*<0.05, right thigh circumference vs. left thigh circumference in women; ^c^*P*<0.05, right handgrip strength vs. left handgrip strength in men; ^d^*P*<0.05, right handgrip strength vs. left handgrip strength in women

[Caption] There were significant differences between men and women in thigh circumference and handgrip strength, and the circumference and strength of the right sides were more likely to be wider and stronger than those of the left sides. In contrast, these differences were not observed in quadriceps muscle thickness.

# Additional Table 2. Sample characteristics by absence or presence of fall injury

| Sample characteristics | No fall injury  (n = 136) | Fall injury  (n = 44) | *P*-value |
| --- | --- | --- | --- |
| Age, years | 69 (62–75) | 70 (65–79) | 0.21 |
| Male sex, n (%) | 99 (72.8) | 28 (63.6) | 0.25 |
| Duration of dialysis, years | 5.1 (2.3–9.3) | 6.5 (2.5–10.6) | 0.40 |
| Body mass index (kg/m^2^) | 21.9 (19.6–24.8) | 20.7 (18.9–24.0) | 0.14 |
| Follow-up period, days | 365 (365–365) | 233 (153–302) | <0.001 |
| Dialysis prescription |  |  |  |
| HD duration of 3.5 h/4.0 h/>4.0 h, n | 1/119/16 | 2/40/2 | 0.17 |
| Fluid removal, L | 2.4 (1.9–3.1) | 2.5 (2.1–3.1) | 0.42 |
| Antihypertensive drug, 0, 1, 2, or 3 or more, n | 20/16/25/30 | 3/4/10/9 | 0.62 |
| Benzodiazepine drugs, n (%) | 28 (21) | 8 (18) | 0.79 |
| Any treatments for intradialytic hypotension, n (%) | 25 (18) | 11 (25) | 0.18 |
| Comorbidities |  |  |  |
| Diabetes mellitus, n (%) | 21 (15) | 13 (30) | 0.001 |
| Stroke, n (%) | 13 (22) | 10 (17) | 0.047 |
| Ischemic heart disease, n (%) | 45 (33) | 22 (50) | 0.049 |
| Serum albumin (g/dl) | 3.8 (3.6–3.9) | 3.7 (3.4–3.9) | 0.07 |
| HDL-cholesterol (mg/dl) | 41 (35–51) | 41 (33–55) | 0.80 |
| LDL-cholesterol (mg/dl) | 78 (64–100) | 79 (62–94) | 0.69 |
| Triglyceride (mg/dl) | 87 (61–135) | 85 (60–120) | 0.26 |
| Uric acid (mg/dl) | 7.0 (6.0–8.1) | 6.6 (5.7–7.4) | 0.06 |
| CRP (mg/dl) | 0.11 (0.05–0.25) | 0.15 (0.05–0.51) | 0.13 |
| BUN (mg/dl) | 65 (57–77) | 61 (53–69) | 0.06 |
| Creatinine (mg/dl) | 11.2 (9.4–12.6) | 9.5 (8.0–10.4) | <0.001 |
| Single pool Kt/Vurea | 1.44 (1.36–1.59) | 1.41 (1.29–1.59) | 0.23 |
| Ca (mg/dl) | 8.7 (8.3–9.1) | 8.8 (8.2–9.3) | 0.75 |
| P (mg/dl) | 5.4 (4.6–6.3) | 5.3 (4.3–5.9) | 0.13 |
| Casual blood glucose, (mg/dl) | 125 (105–154) | 133 (109–192) | 0.45 |
| Intact PTH (pg/ml) | 136 (78–194) | 139 (73–237) | 0.97 |
| β_2_ microglobin (mg/l) | 26.3 (22.9–29.5) | 26.9 (22.4–29.6) | 0.72 |
| Hemoglobin (g/dl) | 10.7 (10.3–11.2) | 10.9 (10.4–11.4) | 0.30 |
| Quadriceps muscle thickness (cm) | 4.2 (3.6–5.1) | 3.4 (3.1–4.2) | <0.001 |
| Thigh circumference (cm) | 43 (40–47) | 41 (37–44) | 0.007 |
| Handgrip strength (kg) | 25 (21–32) | 20 (16–23) | <0.001 |

Abbreviations: n, number; HDL, high-density lipoprotein; LDL, low-density lipoprotein; CRP, C-reactive protein; BUN, blood urea nitrogen; PTH, parathyroid hormone

Data are expressed as the median (interquartile range) or number (percentage)

[Caption] Patients with fall injury were more likely to be diabetes, history of stroke, and history of ischemic heart disease, and those were more likely to have lower creatinine, quadriceps muscle thickness, thigh circumference and handgrip strength.
