# Supplementary material for: Quantitative sonographic assessment of quadriceps muscle thickness for fall injury prediction in patients undergoing maintenance hemodialysis: an observational cohort study
Source: BMC Nephrol. 2021 May 22;22:191. doi: 10.1186/s12882-021-02347-5 (PMC8140437; doi:10.1186/s12882-021-02347-5)
Supplement: Supplementary file 2 — Additional file 2: Figure. Kaplan–Meier curves for fall injury in quadriceps muscle thickness, thigh circumference, and handgrip strength. [file 12882_2021_2347_MOESM2_ESM.pptx]

## Slide 1
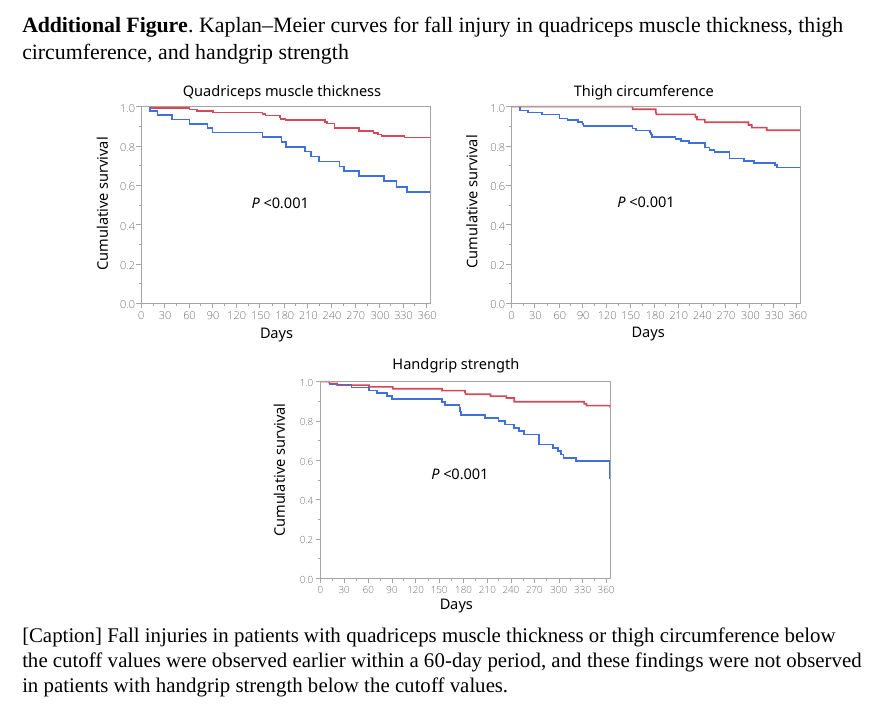

Additional Figure. Kaplan–Meier curves for fall injury in quadriceps muscle thickness, thigh circumference, and handgrip strength
Quadriceps muscle thickness
Thigh circumference
Cumulative survival
P <0.001
P <0.001
Cumulative survival
Days
Days
Handgrip strength
Cumulative survival
P <0.001
Days
[Caption] Fall injuries in patients with quadriceps muscle thickness or thigh circumference below the cutoff values were observed earlier within a 60-day period, and these findings were not observed in patients with handgrip strength below the cutoff values.
